# Conservative Management of Spontaneous Left Main Coronary Artery Dissection (SCAD) Triggered by Emotional Stress in the Late Postpartum Period: Case Report and Pathophysiology

**DOI:** 10.3390/pathophysiology29040047

**Published:** 2022-10-26

**Authors:** Jaksa Zanchi, Dino Miric, Lovel Giunio, Anteo Bradaric Slujo, Mislav Lozo, Duje Erceg, Duje Orsulic, Josip A. Borovac

**Affiliations:** 1 Division of Interventional Cardiology, Clinic for Heart and Vascular Diseases, University Hospital of Split (KBC Split), Spinciceva 1, 21000 Split, Croatia; 2 Department of Pathophysiology, University of Split School of Medicine, Soltanska 2, 21000 Split, Croatia; 3 Clinic for Anaesthesiology, Reanimatology and Intensive Care, University Hospital of Split (KBC Split), Spinciceva 1, 21000 Split, Croatia; 4 Department of Cardiac Surgery, University Hospital of Split (KBC Split), Spinciceva 1, 21000 Split, Croatia

**Keywords:** cardiogenic shock, conservative management, emotional stress, left main coronary artery, postpartum, spontaneous coronary artery dissection, SCAD

## Abstract

A spontaneous coronary artery dissection (SCAD) during the postpartum period is a serious medical emergency and the most important non-atherosclerotic cause of coronary artery disease (CAD) in this population. While conservative management is recommended in most SCAD scenarios, cases complicated by hemodynamic instability or cardiogenic shock are particularly challenging and might be amenable only with invasive percutaneous or cardiothoracic surgical management. Herein, we present a case of a 35-year-old otherwise healthy woman that suffered an intense emotional stress event and was subsequently admitted with crushing chest pain to the emergency department. The initial electrocardiogram showed dynamic changes suggesting anterolateral ST-elevation myocardial infarction. She gave birth to a healthy child 3 months before the current presentation. Diagnostic angiography found no occlusive CAD but instead an extensive intramural hematoma originating from the left main artery dissection and extending to the whole left coronary circulation was observed. Hemodynamic instability and hypotension soon followed, and the patient went into cardiogenic shock. The heart team opted for conservative and supportive intensive care management without surgical or percutaneous intervention. This decision ultimately led to the successful extubation of the patient and the achievement of hemodynamic stability. The patient was eventually safely discharged home without any permanent disability.

## 1. Introduction

Spontaneous coronary artery dissection (SCAD) is an important non-atherosclerotic cause of acute coronary syndrome (ACS), especially among younger female patients without traditional cardiovascular risk factors. The latest data show that extra coronary arteriopathies, such as fibromuscular dysplasia, might be associated with a predilection for SCAD [1]. SCAD is known to occur during the third trimester of pregnancy and in the early postpartum period, with hormonal changes and hemodynamic stress during pregnancy and puerperium potentially representing precipitating factors leading to initial intimal vessel rupture [2,3].

Angiographic findings of SCAD are characterized by the presence of false lumen due to intimal disruption (intimal tear) and formation of intramural hematoma due to bleeding originating from vasa vasorum. These changes are often accompanied with periadventitial inflammation with eosinophil predominance [4]. Advanced intracoronary imaging revealed that intramural hematoma is associated with the false lumen, while partial or complete thrombosis may be acutely present in both true and false lumen [5,6]. More recent studies suggest that an “outside-in” pathophysiological mechanism is at play in most of patients with SCAD, meaning that the initial event is intraparietal bleeding, thus suggesting that these patients might be at greater relative risk of bleeding than thrombosis [7]. This has practical repercussion, as an overly aggressive antithrombotic regimen, such as dual antiplatelet therapy (DAPT) prescription at discharge, might favor bleeding events in the future and be associated with worse clinical outcomes, as recent registry data has shown [8]. Similarly, among SCAD patients treated conservatively in the DIssezioni Spontanee COronariche (DISCO) registry, prescription of DAPT at discharge was associated with significantly worse 1-year outcomes compared to those prescribed with single antiplatelet therapy (SAPT) [9]. Therefore, the latest data suggest that one antiplatelet agent following the SCAD event would be the most appropriate mode of treatment; however, future randomized studies comparing different antithrombotic strategies are warranted.

In the acute and subacute setting, most patients with SCAD can be managed conservatively, since the dissected vessel usually self-repairs and spontaneously heals over a few weeks or months. In contrast to this, results of revascularization with percutaneous coronary intervention (PCI) and coronary artery bypass grafting (CABG) are suboptimal and are often associated with an increased rate of complications and adverse in-hospital events compared to optimal conservative management [10]. However, the interventional or cardiosurgical approach must be considered as an option in complicated SCAD cases such as those that involve the left main stem or are characterized by ongoing active ischemia (chest pain, hemodynamic instability, or sustained ventricular arrhythmias).

Herein, we present a case of a young woman without cardiovascular risk factors diagnosed with SCAD during the delayed postpartum period whose clinical presentation and symptoms were provoked by intense emotional stress. Furthermore, her SCAD case had several high-risk features, such as the dissection of the left main stem propagating to the whole left coronary artery tree and the onset of hemodynamic instability with progression to cardiogenic shock. Finally, we show how careful selection of conservative management yielded the successful recovery of the patient in this complicated SCAD scenario.

## 2. Case Report

A 35-year-old female without known chronic illnesses, allergies, and cardiovascular risk factors was admitted to the emergency department with symptoms of crushing substernal chest pain with propagation to the throat and accompanied with electrocardiographic signs of anterolateral ST-segment elevation myocardial infarction (STEMI) (elevation in leads D1, aVL, V2, and V3 with reciprocal horizontal ST depressions in D2, D3, and aVF). The important differential diagnosis of STEMI is provided in Table 1.

The patient experienced significant emotional distress due to a family feud shortly before the onset of symptoms and was in a delayed postpartum period, as she gave healthy childbirth 3 months earlier. Physical examination revealed no heart murmurs upon auscultation and no pathological breathing sounds. Furthermore, she was afebrile, with a heart rate of 104 beats per minute, peripheral saturation of 93% on pulse oximetry, and initial arterial blood pressure of 103/73 mmHg. She had normally palpable peripheral pulses on both sides. The patient was immediately transferred to the coronary care unit (CCU) for STEMI pathway, and she was perorally loaded with 300 mg of acetylsalicylic acid and 180 mg of ticagrelor along with 5000 IU of unfractionated heparin intravenously. Her initial laboratory workup revealed slightly elevated D-dimer levels (0.53 mg/L, upper range reference cut-off value of 0.50 mg/L) with high-sensitivity cardiac troponin I (hs-cTnI) and C-reactive protein within normal limits (1.8 ng/L and 4.6 mg/L, respectively).

**Figure 1:**
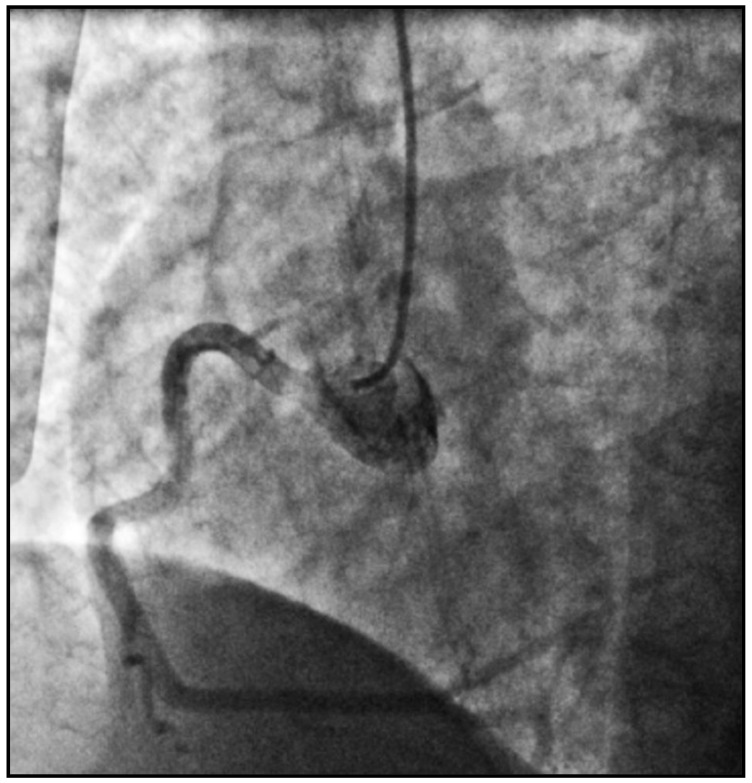
A coronary angiogram of the right coronary artery (RCA).

She was immediately transferred to cath lab from CCU, and an emergent coronary angiography was performed by intubating a right coronary artery (RCA), with the 6F diagnostic catheter showing a dominant high-caliber vessel giving off posterolateral (PL) and posterodescending (PD) branches with normal TIMI-3 flow and no signs of obstructive coronary disease (Figure 1).

**Figure 2:**
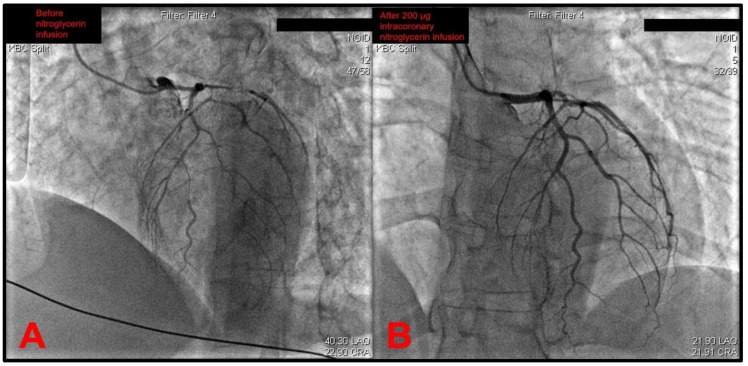
(**A**) An emergent coronary angiography revealed no atherothrombotic occlusions and a gracile aspect of the whole left coronary tree with most probable differential diagnoses of severe diffuse coronary vasospasm or extensive intramural hematoma due to a spontaneous coronary artery dissection (SCAD)—in this case originating from the left main stem. (**B**) Distal coronary flow significantly improved after an intracoronary infusion of 200 micrograms of nitroglycerin. Note: operator name, patient information, and procedure date have been de-identified from the original images.

The catheter was then exchanged, and the left coronary artery (LCA) was later intubated. This revealed a gracile left coronary circulation characterized by the narrow-caliber LM that was prone to spasm and a narrow caliber LAD that featured a slow flow as well as the angiographical picture of an intramural hematoma due to a spontaneous coronary artery dissection (SCAD) originating from the left main stem (Figure 2A). A differential diagnosis of severe diffuse coronary vasospasm was considered as well, given the narrow and gracile caliber of all vessels originating from the left main stem. The circumflex artery (Cx) was found to have a slow flow, and it also featured severe diffuse spasms. Furthermore, it also gave off a strong marginal branch that had slow flow and had a significantly larger diameter than the circumflex. The intermediate branch was also observed to be characterized by gracile morphology, slow flow, and subocclusion of the proximal segment. An intracoronary infusion of 200 μg of nitroglycerin significantly recovered the distal flow through the left coronary tree (Figure 2B). Although it would be an appropriate step to perform intracoronary imaging to confirm the suspected SCAD diagnosis, this was not performed due to a lack of dedicated devices in the cath lab at the time.

Movies depicting the selective coronary intubation of RCA and LCA are shown in Movies S1 and S2, respectively, while a movie showing significant improvement in the distal flow of the left coronary tree following the intracoronary administration of 200 μg nitroglycerin is provided in Movie S3. While performing diagnostic angiography, the patient’s condition abruptly deteriorated, with hypotension, progression to pulmonary edema, and cardiogenic shock. As can be seen in Movie S2, occlusion of distal LAD and a large marginal obtuse branch was observed, thus suggesting the extension of the dissection from the LM to distal vessels, and this might have been associated with the first contrast injection. A critical care anesthesiologist was called, and the patient was immediately endotracheally intubated and placed on mechanical ventilation with the administration of intravenous dobutamine and boluses of furosemide. A multidisciplinary heart team was quickly summoned, and the decision was made to continue conservative rather than invasive or surgical management of SCAD.

The patient was, therefore, transferred to the cardiosurgical intensive care unit (CICU) for further clinical evaluation and potential provision of extracorporeal membrane oxygenation (ECMO). Her initial hs-cTnI level at admission to CICU was 132,470 ng/L, while her urine output was about 500 mL. Advanced critical care measures were continued, with mechanical ventilation and the concomitant placement of arterial line, central venous catheter, and pulmonary catheter. She was placed on levosimendan and noradrenaline drip, while supportive treatment included analgesia and sedation in continuous infusion with stress ulcer prophylaxis and broad-spectrum antibiotic administration. The patient remained hemodynamically stable, and on the next day she was successfully weaned from mechanical ventilation and was able to achieve adequate oxygen saturation on ambient air. Similarly, after vasopressor and inotrope support was discontinued, she was able to sustain normal hemodynamics. Her hs-cTnI value at discharge from CICU was 29,821 ng/L. She was then transferred to the post-intensive care unit, with peroral therapy including amoxicillin-clavulanate, pantoprazole, acetylsalicylic acid, clopidogrel, bisoprolol, atorvastatin, furosemide, and bromocriptine with the administration of subcutaneous enoxaparin in prophylactic antithrombotic dose.

The patient’s follow-up chest X-ray was performed, which showed no acute infiltrates and was otherwise normal, except for the small residual pleural effusion in the right costodiaphragmatic recess. Upon stabilization of her clinical condition and 11 days after the index event, a follow-up coronary angiography was performed, which showed normal LM, LAD, and RCA with established TIMI-3 flow, while there was an intermediate 50% stenosis found in the circumflex artery for which a decision was made to treat conservatively. In-hospital transthoracic echocardiographic examination prior to discharge showed an ischemic remodeling of the left ventricle (LV) with residual distal septal, anteroapical, and apical hypokinesia with grade 2 diastolic dysfunction and normal systolic function of the right ventricle. The LV ejection fraction (LVEF) was mildly reduced (45% to 50%). No septal shunting was observed, and valvular function was normal.

The total length of the patient’s hospital stay was 17 days, and she was discharged in a stable clinical condition. Her treatment at discharge consisted of acetylsalicylic acid (100 mg OD), clopidogrel (75 mg OD for the next 12 months), pantoprazole (20 mg OD), atorvastatin (80 mg OD), and bisoprolol (1.25 mg OD). In the last outpatient follow-up visit, her cardiac systolic function improved to >50%; however, no repeat angiographies were later performed at our institution. Similarly, details on further fibromuscular dysplasia work-up were unavailable in our records.

## 3. Discussion

From the pathophysiological perspective, SCAD is largely caused by underlying non-atherosclerotic systemic arteriopathies, such as fibromuscular dysplasia (FMD), while it is often precipitated by emotional or physical stressors and circumstances, such as labor and delivery [11]. Pathohistological and intracoronary imaging studies show that the origin of spontaneous dissection occurs in the outer third of the tunica media of the coronary vessel, with this space being occupied by the intramural hematoma compressing the true lumen and causing obstructed coronary blood inflow and subsequent myocardial infarction, with or without the presence of intimal tear [12]. SCAD most frequently occurs in patients with few or no traditional cardiovascular risk factors, with a strong predilection for women of childbearing age in whom it might constitute even up to 35% of ACS events under the age of 50, while at the same time, it is considered to be the most common cause of pregnancy-associated MI [12]. Most SCAD cases affect the territory of a left anterior descending artery and its branches, followed by circumflex and obtuse marginal branches, and right coronary circulation, while dissection originating from the left main artery is least frequent and makes up 1.2% to 4% of all SCAD cases [11]. Similarly, mid to distal segments of coronary arteries are usually most affected.

A Canadian SCAD cohort study encompassing 22 centers in North America showed that women experiencing SCAD during peripartum have worse in-hospital outcomes and more severe/complex lesions than their non-peripartum counterparts, while emotional and physical stress were reported as precipitants in 50.3% and 28.9% of cases, respectively [13]. The vast majority of pregnancy-associated SCAD events occur during the third trimester or early postpartum period, although cases occurring in early pregnancy and up to a year after childbirth have been reported previously [14]. The gold standard diagnostic method for SCAD is coronary angiography, while the most common angiographic appearance is a mild to severe diffuse stenosis, also classified as type 2A SCAD lesion, as shown in our case [14]. Of note, Saw et al. provided the angiographic classification of SCAD lesions, arguing that optical coherence tomography (OCT) or intravascular ultrasound (IVUS) should be the true gold standard to diagnose SCAD, since plain coronary angiogram does not allow for the visualization of the arterial wall [15]. The pathognomonic appearance of multiple radiolucent lumens on angiography may be absent in as many as 70% of cases, therefore, operators performing angiography should be aware of various angiographic SCAD variants to improve diagnosis and use intracoronary imaging (OCT, IVUS) when uncertain [16].

In this case report, an otherwise healthy female presented with SCAD precipitated by intense emotional stress during the late postpartum period (6 weeks to 12 months), with the dissection originating from the left main stem and complicated by cardiogenic shock. Although high-risk features, such as the presence of hemodynamic instability, involvement of the left main stem, or proximal LAD or extensive length of the lesion, might be legitimate triggers for percutaneous or surgical revascularization, herein we showed how conservative management of such complex clinical conditions yielded a favorable outcome without further neurological or cardiovascular sequelae. Of importance, even with technically successful PCI, important complications such as the extension of dissection might occur in 25% to 60% of cases, with long-term patency of the vessel achieved in only about a third of all such cases [14]. Similarly, a useful teaching point should be emphasized: in cases when interventional cardiologists suspect coronary dissection, such as LM SCAD in this case, it is important to avoid performing additional angiographic projections and contrast injections, since this can enhance the expansion of hematoma distally.

**Figure 3:**
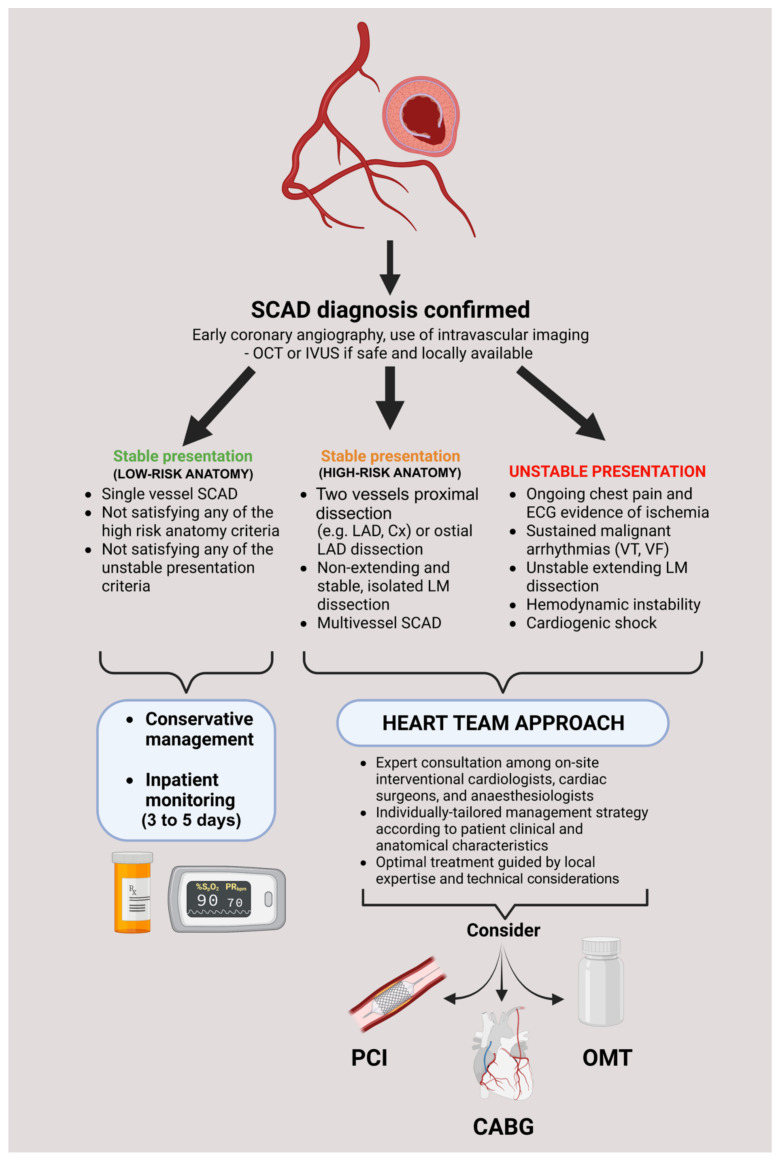
A decision tree in approaching confirmed SCAD cases during coronary angiography. Abbreviations: CABG: coronary artery bypass grafting; Cx: circumflex artery; IVUS: intravascular ultrasound; LAD: left anterior descending artery; LM: left main; OCT: optical coherence tomography; OMT: optimal medical therapy; PCI: percutaneous coronary intervention; VF: ventricular fibrillation; VT: ventricular tachycardia.

Recent data suggest that conservative management in SCAD should prevail in most cases, as such an approach has been associated with lower target vessel revascularization rates and non-inferior clinical outcomes compared to invasive management [17,18]. It should be highlighted that no official guidelines or sufficient data exist on the optimal management of SCAD due to a lack of dedicated randomized studies, and most of the recommendations are based on the consensus of experts or position papers such as by Adlam and colleagues, published on the behalf of the European Society of Cardiology [10]. As exemplified in our case, the decision to treat SCAD conservatively in the setting of cardiogenic shock might be a double-edged sword and difficult to justify in some clinical scenarios; however, we firmly hold that management decisions should be thoroughly discussed by the multidisciplinary heart team and tailored to an individual patient by meticulously acknowledging relevant angiographic and clinical factors. A proposed algorithm of treatment/decision pathway in SCAD cases is provided in Figure 3.

Finally, patients with SCAD, predominantly women, should be advised to perform further work-up for associated conditions or factors. These include FMD, as recent studies implicate the close genetic association between these two entities, while other vascular territories should also be investigated as per clinical indication since SCAD is associated with a high prevalence of extracoronary dissections, inherited arteriopathies, connective tissue disorders, and aneurysms [12,19,20].

## 4. Conclusions

SCAD is an important nonatherosclerotic cause of ACS in women of childbearing age without traditional cardiovascular risk factors and should raise clinical suspicion particularly if the patient’s history has a context of intense psychophysical stress that occurs during pregnancy or in the postpartum period. Here, we reported a case in which SCAD extending from the left main stem further complicated by hemodynamic instability and cardiogenic shock was successfully managed conservatively in a female patient during the late postpartum period by utilizing a multidisciplinary “heart team” approach. These sorts of patients should be carefully monitored through regular cardiology referrals, and a low threshold for genetic testing and exclusion of other vascular abnormalities should be individually employed by adhering to postulates of precision medicine.

## Figures and Tables

**Table 1:** Important differential diagnoses to consider among women of childbearing age without apparent history and/or risk factors for cardiovascular disease.

ECG Signs of ST-Elevation Myocardial Infarction (STEMI) or Non-ST-Elevation Myocardial Infarction (NSTEMI) + Ongoing Chest Pain Symptoms
Coronary atherothrombosis due to plaque rupture or erosion
Spontaneous coronary artery dissection (SCAD)
Coronary artery vasospasm
Coronary embolism
Takotsubo cardiomyopathy
Microvascular coronary disease (MVD)
Myocardial infarction in non-obstructed coronary arteries (MINOCA)

## Data Availability

Not applicable.

## Supplementary Materials

The following supporting information can be downloaded at: https://www.mdpi.com/article/10.3390/pathophysiology29040047/s1.
